# PIK3CD correlates with prognosis, epithelial–mesenchymal transition and tumor immune infiltration in breast carcinoma

**DOI:** 10.1007/s12672-023-00805-0

**Published:** 2023-10-20

**Authors:** Wenxing He, Haoyi Zhang, Hong Cheng, Jianfeng Wen, Dongmei Li

**Affiliations:** 1 Breast Cancer Center, Jiangxi Cancer Hospital of Nanchang Medical College, No. 519 East Beijing Road, Nanchang, 330029 People’s Republic of China; 2 School of Public Health, Nanchang University, Nanchang, 330006 China; 3 Hospital 908 of the Joint Support Force of the Chinese People’s Liberation Army, Nanchang, 330002 China; 4 Jiangxi Key Laboratory of Translational Research for Cancer, Jiangxi Cancer Hospital of Nanchang Medical College, No. 519 East Beijing Road, Nanchang, 330029 Jiangxi China; 5 Nanchang County Maternal and Child Health Hospital, Nanchang, 330200 People’s Republic of China

**Keywords:** Breast cancer, PIK3CD, CircRNA, Epithelial–mesenchymal transition, Prognosis, Tumor immune infiltration

## Abstract

**Background:**

Breast carcinoma (BRCA) is one of the most common, fatal, and aggressive cancers, with increasing morbidity that has a major impact on human health. PIK3CD appears to have important roles in the beginning and advancement of various forms of human cancer, according to mounting data. However,the particular role and mechanism of PIK3CD in BRCA remains not fully identified.

**Methodology:**

The Cancer Genome Atlas (TCGA, https://portal.gdc.cancer.gov/), Genotype-Tissue Expression (GTEx) data and the UCSC Xena browser (https://xenabrowser.net) data were used in this study’s initial pan-cancer analysis of PIK3CD expression and prognosis. Circular RNAs (circRNAs) that regulated the expression of PIK3CD were subsequently found using a combination of in silico investigations of expression, correlation, and survival. Measurements of PIK3CD expression and an analysis of the in vitro function of PIK3CD in BRCA cells were performed using real-time RT-PCR, Western blotting and Transwell assays.

**Results:**

In BRCA GLI2, RAB32, LAMB1, MGAT2, ITGA8, CHF, COL6A3 and PRRX1-miR-30b-5p axis was identified as the most likely upstream CircRNA-related route of PIK3CD. PIK3CD was correlated with the expression of EMT markers. The PIK3CD cDNA improved the capacity for invasion and migration. The expression of PIK3CD was linked to some of the m1A/m5C/m6A regulators. Additionally, it was discovered that the expression of PIK3CD was found to be highly connected to the expression of immunological checkpoints, immune cell biomarkers, and tumor immune cell invasion.

**Conclusions:**

Our findings reveal that PIK3CD expression is associated with prognosis, EMT, and tumor immune infiltration in BRCA patients.

## Introduction

The most frequent primary cancer and the second greatest cause of deaths due to cancer in women in the US is breast cancer (BRCA).Breast cancer is still difficult to prevent globally because it is a multi-step procedure that involves many types of cells. One of the best ways to avoid breast cancer is through early diagnosis [1]. Several risk factors, including the immune system, have been connected to the beginning and development of BRCA. The prognosis for people with BRCA remains poor despite the enormous advances made in the fields of diagnosis, treatment, and prognosis. Thus, it is crucial to find viable prognostic biomarkers or create efficient treatment targets for BRCA.

The phosphoinositide 3-kinase (PI3K) is one of the most prevalent regulatory mechanisms in a range of human malignancies. Based on their substrate specificity and mammalian structural characteristics, PI3K is split into three types. The PI3K subfamily of class I appears to be the one most associated with human malignancies. According to their adaptors, Class I PI3K is further separated into subclasses IA and IB. Class IA catalytic isoforms p110α, p110β and p110δ are encoded three distinct genes termed PIK3CA, PIK3CB and PIK3CD. The functions and fundamental mechanisms involving PIK3CD in cancer, however, are poorly understood [2].

The epithelial–mesenchymal transition (EMT) can promote metastasis in a variety of cancer types. EMT was linked to the exclusion of immune cells important in the immune reaction to cancer as well as the expression of several immunosuppressive cytokines. There may be a link between EMT and tumor immune infiltration in cancer [3].

In our study, we first examined the expression and survival of PIK3CD in diverse human cancers. Following that, the CircRNA-related regulation of PIK3CD in BRCA was investigated. Subsequently, we examined the connection between PIK3CD expression and influx of immunological cells, biological markers, or immunological checkpoints in BRCA. These results suggest that PIK3CD is associated with tumor immune infiltration and prognosis in BRCA individuals.

## Material and methods

### Download, processing and analysis of TCGA data

The mRNA expression data of 24 tumor types (BLCA, BRCA, CHOL, COAD, ESCA, BMLGG, HNSC, KICH, KIRC,KIRP, LAML, LIHC, LUAD, LUSC, OV, PAAD, PRAD, SKCM, STAD, STES, TGCT, THCA, UCEC and WT) were retrieved from the TCGA database (https://genome-cancer.ucsc.edu/), Genotype-Tissue Expression (GTEx) data and the UCSC Xena browser (https://xenabrowser.net) and then differential expression analysis for PIK3CD was conducted using the R package limma after normalization. Statistical significance was determined by a p value of < 0.05.

### Analysis of the GEPIA database

GEPIA (http://gepia.cancer-pku.cn/) is a digital platform based on TCGA and Genotype-Tissue Expression (GTEx) data for cancer and normal gene expression profiling and interactive analytics. PIK3CD and circRNA expression in various types of human cancer were determined using GEPIA. The p value of < 0.05 was considered statistically significant. GEPIA was utilized to conduct PIK3CD survival study in different cancer types, including OS and RFS. GEPIA was also used to analyze the predictive relevance of putative BRCA circRNAs. The logrank p value of < 0.05 was used to evaluate statistical significance. Furthermore, the expression connection of PIK3CD with immunological checkpoints in BRCA was evaluated using the GEPIA database. The selection criteria for identifying it as statistically significant were R > 0.1 and p value < 0.05.

### Analysis using the Kaplan–Meier plotter

As initially noted, the Kaplan–Meier plotter (http://kmplot.com/analysis/), an online resource able to evaluate gene or miRNA influence on survival in over 20 cancer types, including BRCA, was utilized to perform survival analysis for miRNA in BRCA. Log rank p value of < 0.05 was regarded as statistically significant.

### Analysis of the StarBase database

StarBase is a database for miRNA research (http://starbase.sysu.edu.cn/). In BRCA, starBase was used to conduct an expression analysis of correlation for miRNA-PIK3CD, circRNA-miRNA, or circRNA-PIK3CD. StarBase was also used to examine miRNA-30b-5p expression in BRCA and normal controls. Furthermore, starBase was employed to anticipate candidate circRNAs that might bind to miRNA-30b-5p.

### Prediction of candidate miRNAs

Several target gene prediction programs, including PITA, RNA22, miRmap, microT, miRanda, PicTar and Tar-getScan, anticipated upstream binding miRNAs of PIK3CD. The following analyses included only anticipated miRNAs that showed often in more than two programs, as previously noted. These expected miRNAs were considered candidate miRNAs of PIK3CD.

### Cell culture

The Shanghai Institute of Cell Biology’s and the Chinese Academy of Sciences’ Cell Banks provided the breast cancer MCF-7 cells and MDA-MB-231 cells for this study. The cell lines were grown in Roswell Park Memorial Institute (RPMI)-1640 medium (Invitrogen) containing 10% fetal bovine serum (Invitrogen) and antibiotics (100 g/mL streptomycin and 100 U/mL penicillin) at 37 °C and 5% CO2 in a humidified tissue culture chamber. Cells from the period of logarithmic growth were employed in further tests.

### Lentivirus production and transduction

The NC (negative control) mRNA precursor sequences and the PIK3CD sequences (cDNA) were amplified and inserted into the pLV3 vector, both from GeneChem in Shanghai, China. For virus production, HEK293T cells were transfected with the packaging vectors pGag/Pol, pRev and pVSV-G in combination with the recombinant lentiviral plasmids. After 48 h of transfection, the lentivirus-containing medium was recovered and filtered. The viral supernatant containing polybrene was then introduced to the breast cancer MDA-MB-231 cells and MCF-7 cells to cause infection. The transduced cells were then chosen for 14 days of puromycin to identify those that were consistently producing the constructs. It was determined how much PIK3CD was present in these stable cells using quantitative real-time PCR [2].

### RT-PCR in real time

Total RNA was isolated from cultivated cells using the Trizol reagent (Invitrogen) according to the instructions supplied by the manufacturer. SYBR®Green (Thermo Fisher Scientific) was used in quantitative RT-PCR to measure the mRNA levels of the listed genes.Internal standards for mRNA and miRNA were GAPDH and U6, respectively. Sequences for the PCR primers were as followed: PIK3CD (FW:5′-CATATGTGCTGGGCATTGGC-3′ RV:5′-TTTCACAGTAGCCCCGGAA C-3′), GAPDH (FW:5′-GAGTCAACGGATTTGGTCGT-3′ RV: 5′-GACAAGCTTCCC GTTCTCAG-3′). U6 (FW:5′-CTCGCTTCGGCAGCACA-3′, RV:5’-AACGCTTCACGAATTTGCGT-3’). Dissociation curve analysis was used to confirm particular product amplification. The 2^−ΔΔCt^ method was used to determine gene expression differences.

### Transwell assay

The migration experiment was conducted by seeding cells in the upper chambers without matrigel using inserts (Costar, Cambridge, MA). In invasion assay (BD Bioscience), the inserts were completely covered with matrigel. Cells were resuspended in FBS-free culture and introduced to the upper Transwell chamber, including Matrigel, while the lower chamber received 800 µl of culture medium with 10% FBS.

After the cells were incubated at 37 ℃ for 24 h, the inserts were fixed with methanol and stained with crystal violet at room temperature for 20 min. The cells were taken away from the upper side of the filter membrane, and the invading cells were stained for 10–15 min at room temperature with a 0.1% crystal violet solution. An inverted microscope was used to take the pictures, and ten random fields' worth of cells were counted. Displayed were the statistical analyses and illustrative pictures. Each group’s experiments were carried out in triplicate.

### Western Blot

Cell lysates were produced using a RIPA lysis solution containing a protease inhibitor. The proteins’ concentration was established using a BCA kit from Beijing, China’s Beyotime Biotechnology, before they were separated on polyacrylamide gels (PAGE) with 10% SDS and transferred onto polyvinylidene difluoride (PVDF) membranes. PIK3CD (1:1000, Zenbio, R382122), E-cadherin (1:1000, Affinity, AF0131), vimentin (1:1000, proteintech, 10,366-1-AP), SNAI1(1:1000, proteintech, 13,099-1-AP) and GAPDH (1:1000, Zenbio, 380,626) were then incubated with the hydrogels at 4 ℃. The membranes were incubated for 45 min with a second antibody (1:4000, proteintech, SA00001-2) in combination with horseradish peroxidase following three TBST washes. We imagined and measured the protein bands.

### TIMER database analysis

TIMER (https://cistrome.shinyapps.io/timer/) is a web server for extensive research of tumor-infiltrating immune cells. TIMER was employed to look into the link between PIK3CD expression and infiltration of immune cells or immune-related checkpoint expression in BRCA. A p value of 0.05 was regarded as statistically significant.

### Statistical analysis

The aforementioned internet database generated the statistical analysis for this study automatically. Data from three or more studies were analyzed using R 4.2.1 and given as mean ± SD. The statistical difference was ascertained using either the Student's t test or a one-way ANOVA. The threshold for statistical significance was set at a p value of 0.05 or logrank p value of 0.05. For the results, *p < 0.05,**p < 0.01 and ***p < 0.001 were regarded as statistically significant.

## Results

### Analysis of PIK3CD expression across tumour types

**Fig. 1:**
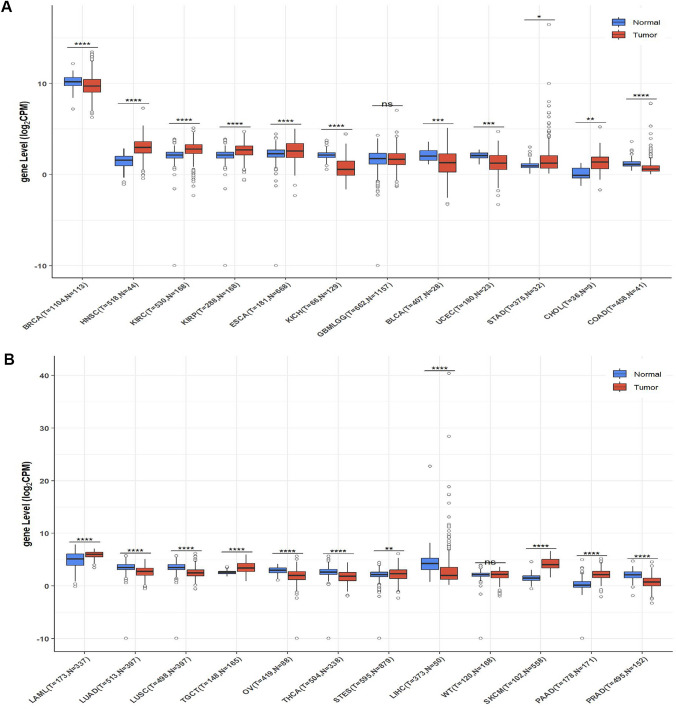
Expression analysis for PIK3CD in multiple tumors. Combining data from the TCGA and GTEx databases, we examined the levels of PIK3CD mRNA expression in 24 different types of human tumors and normal tissues. *p value < 0.05; **p value < 0.01; ***p value < 0.001

To investigate the potential functions of PIK3CD in carcinogenesis, we first examined its expression in 24 different kinds of human tumors. As shown in Fig. 1, PIK3CD was significantly elevated in 11 tumor types, including ESCA, HNSC, KIRC, KIRP, LAML, PAAD, SKCM, STAD, CHOL, STES, and TGCT, and significantly downregulated in 11 tumor types, including BLCA, BRCA, COAD, KICH, LIHC, LUAD, LUSC, OV, PRAD, THCA and UCEC. However, no significant difference in PIK3CD was seen in GBMLGG or WT.

### PIK3CD prognostic values in human tumors.

**Fig. 2:**
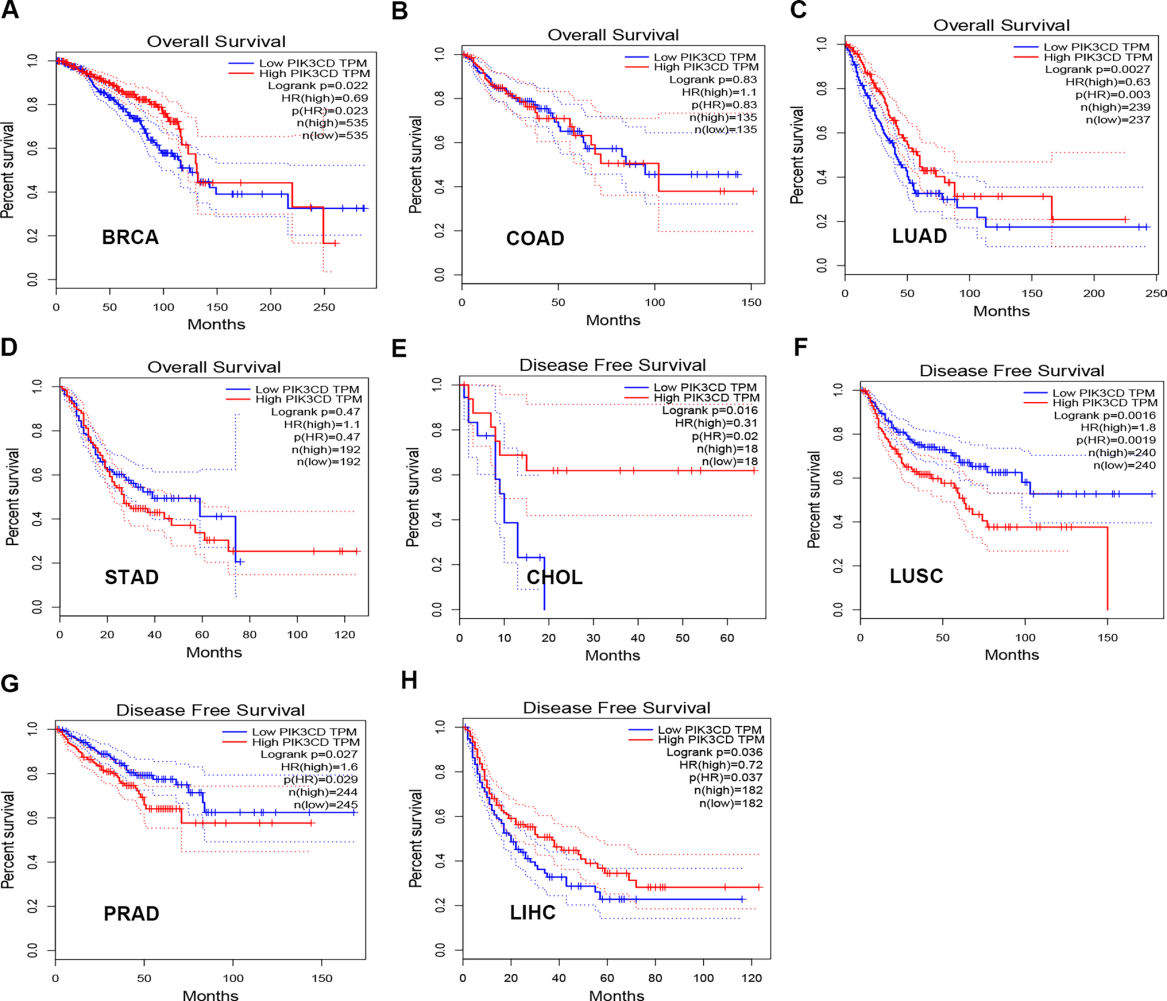
GEPIA assessed the overall survival (OS/RFS) for PIK3CD in diverse human cancers (**A**–**H**). The OS/RFS plot of PIK3CD in BRCA (**A**), COAD (**B**), LUAD (**C**), STAD (**D**), CHOL (**E**), LUSC (**F**), PRAD (**G**) and LIHC (**H**)

The survival analysis for PIK3CD was then performed in BLCA,BRCA, COAD, KICH,LIHC,LUAD,LUSC,OV,PRAD,THCA,ESCA,HNSC,KIRC,KIRP,LAML, PAAD, SKCM,STAD,CHOL, and TGCT. High levels of PIK3CD in LUAD and BRCA were associated with a good prognosis for OS (Fig. 2A–D). Just elevated levels of PIK3CD in PRAD and LUSC patients suggested a poor prognosis for RFS, but higher expression of PIK3CD in CHOL and LIHC patients showed a better prognosis. There was no statistical relevance of PIK3CD in predicting patient outcome in other kinds of cancer (Fig. 2E–H). In patients with BRCA, PIK3CD could be used as a predictive biomarker.

### Prediction and evaluation of PIK3CD's upstream miRNAs

**Fig. 3:**
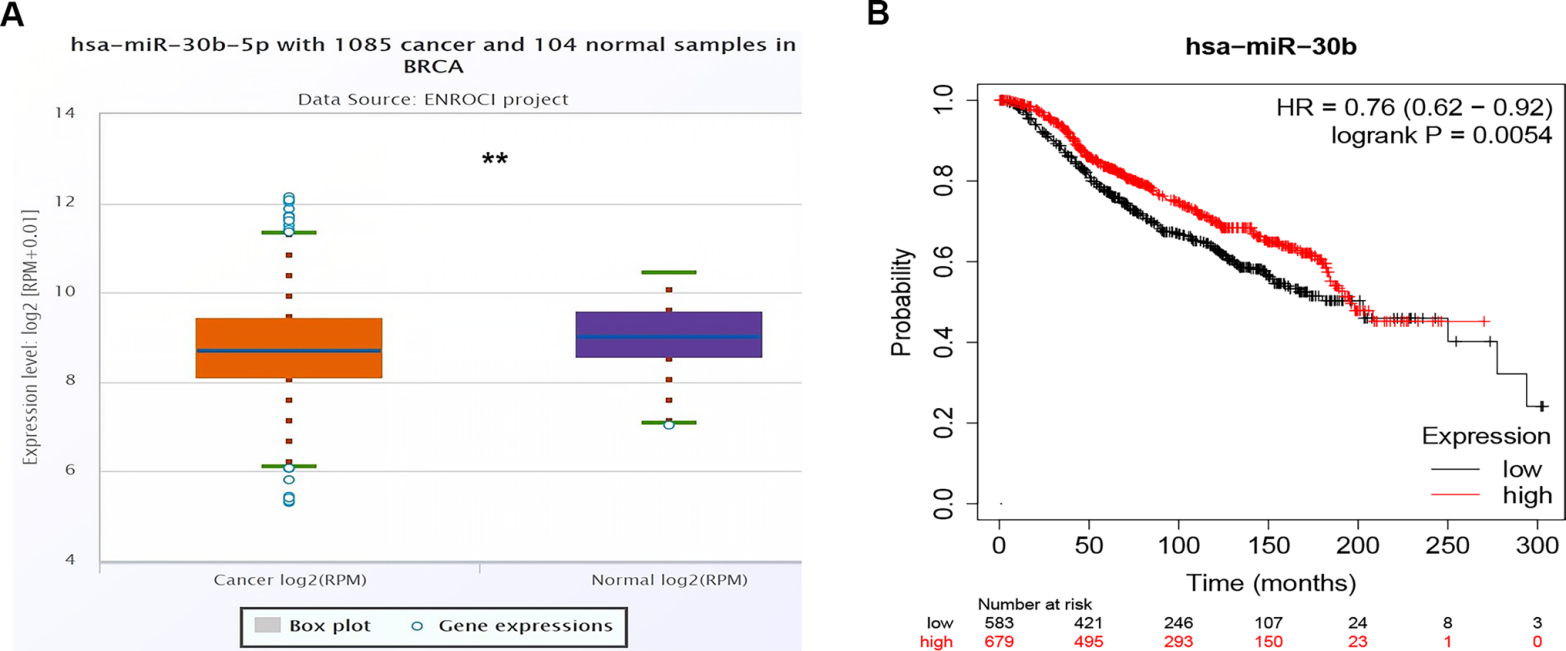
miRNA-30b-5p was markedly downregulated (**A**) in BRCA and miRNA-30b level was positively linked to patients’ prognosis (**B**)

MiRNAs are widely known to be responsible for gene expression regulation. To determine whether PIK3CD was controlled by miRNAs, we predicted upstream miRNAs that could potentially bind to PIK3CD and eventually discovered 6 miRNAs. Based on the competing endogenous RNA (ceRNA) hypothesis, miRNA regulates target gene expression and there should be an adverse correlation between miRNA and PIK3CD.As a result, the expression correlation analysis was carried out. In BRCA, PIK3CD was significantly negatively linked with hsa-miR-30b-5p(|R|= 0.169) and favorably correlated with hsa-miR-30e-5p and hsa-miR-7-5p, as shown in Table 1. PIK3CD and hsa-miR-7-5p have no statistically significant expression correlations. Finally, hsa-miR-30b-5p expression and prognostic significance in BRCA were determined. As shown in Fig. 3A and B, hsa-miR-30b-5p was significantly downregulated in BRCA patients, and its overexpression was associated with a better prognosis. All of these findings point to miRNA-30b-5p as the most likely regulating miRNA of PIK3CD in BRCA.

**Table 1:** The starBase database examined the expression connection between predicted miRNAs and PIK3CD in BRCA

Gene	miRNA	R value	p value
PIK3CD	hsa-miR-30b-5p	− 0.169	1.97E−08
PIK3CD	hsa-miR-30c-5p	− 0.121	6.26E−05
PIK3CD	hsa-miR-30d-5p	− 0.12	7.74E−05
PIK3CD	hsa-miR-30a-5p	− 0.082	7.19E−03
PIK3CD	hsa-miR-30e-5p	0.181	1.87E−09
PIK3CD	hsa-miR-7-5p	0.001	9.83E−01

### Prediction and analysis of upstream circRNAs of hsa-miR-30b-5p

Circular RNAs (circRNAs) have been linked to the progression of cancer [4]. We used bioinformatics to analyze and identify the circRNAs that might probably mediate PIK3CD expression in BRCA.The upstream circRNAs of hsa-miR-30b-5p were then predicted utilizing the starBase database. We investigated the top ten circRNAs. The levels of expression of these circRNAs were subsequently shown to be negatively related to the levels of hsa-miR-30b-5p, which were found to be negatively related to the expression of PIK3CD in BRCA. All of these circRNAs’ expression was linked to PIK3CD (Tables 2 and 3).

**Table 2:** Correlation analysis between CircRNA and miR-30b-5p in BRCA determined by starBase database

miRNA	circRNA	R value	p value
hsa-miR-30b-5p	CFH	− 0.258	6.18E−18
hsa-miR-30b-5p	GLI2	− 0.259	4.59E−18
hsa-miR-30b-5p	RAB32	− 0.245	2.62E−16
hsa-miR-30b-5p	PRRX1	− 0.253	2.60E−17
hsa-miR-30b-5p	LAMB1	− 0.25	5.54E−17
hsa-miR-30b-5p	ITGA5	− 0.278	8.95E−21
hsa-miR-30b-5p	MGAT2	− 0.279	8.68E−21
hsa-miR-30b-5p	COL6A3	− 0.249	8.91E−17
hsa-miR-30b-5p	CD99	− 0.259	3.77E−18
hsa-miR-30b-5p	ITGA8	− 0.278	8.95E−21

**Table 3:** Correlation analysis between circRNAs and PIK3CD in BRCA determined by starBase database

mRNA	circRNA	R value	p value
PIK3CD	CFH	0.479	1.55E−64
PIK3CD	GLI2	0.449	5.25E−56
PIK3CD	RAB32	0.417	8.71E−48
PIK3CD	PRRX1	0.332	6.76E−30
PIK3CD	LAMB1	0.305	3.79E−25
PIK3CD	ITGA5	0.283	8.89E−22
PIK3CD	MGAT2	0.236	2.16E−15
PIK3CD	COL6A3	0.235	2.84E−15
PIK3CD	CD99	0.225	3.77E−14
PIK3CD	ITGA8	0.174	5.99E−09

**Fig. 4:**
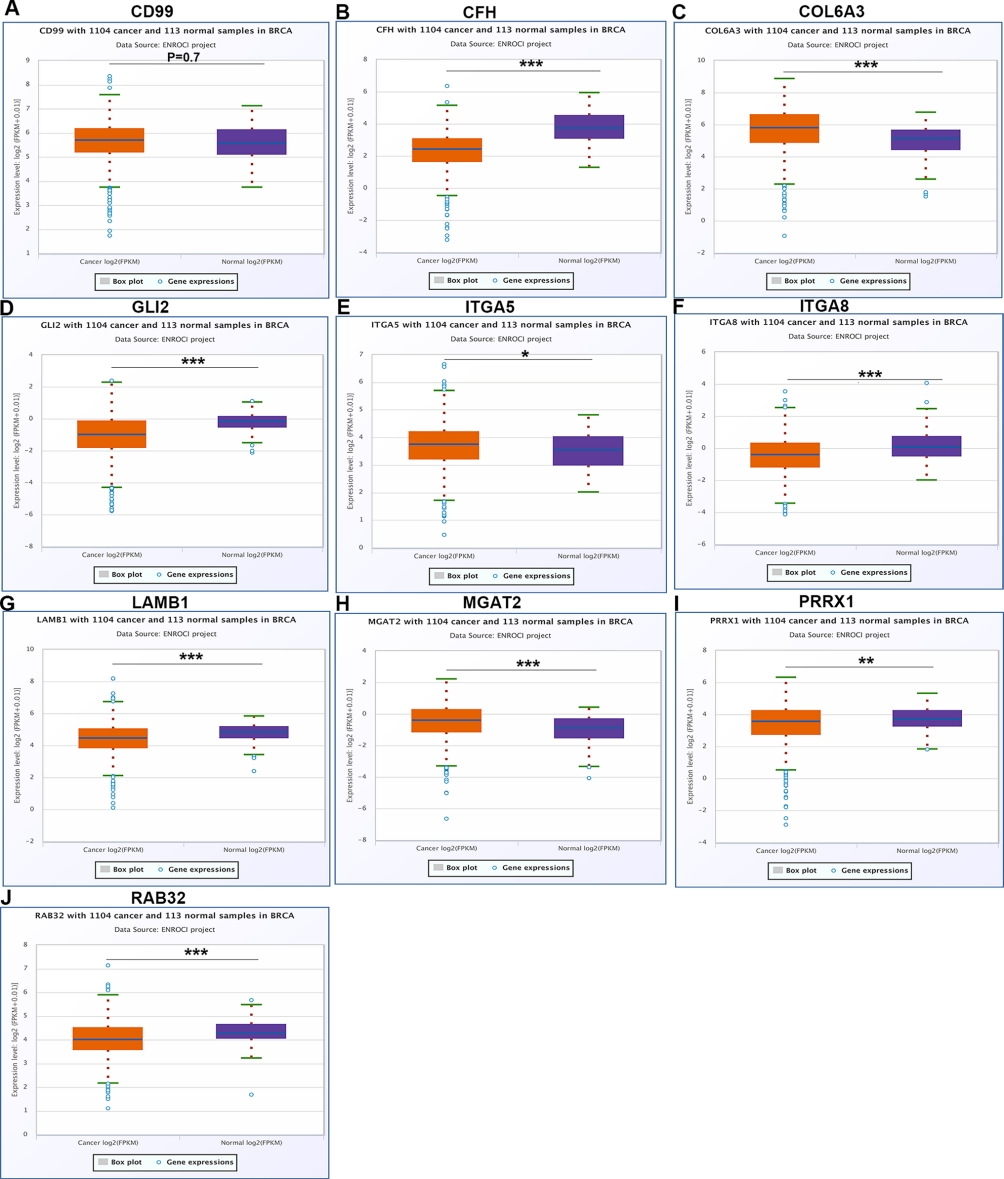
Expression analysis for upstream circRNAs of miR-30b-5p in BRCA.The expression of CD99 (**A**), CFH (**B**), COL6A3 (**C**), GLI2 (**D**), ITGA5 (**E**), ITGA8 (**F**), LAMB1 (**G**), MGAT2 (**H**), PRRX1 (**I**) and RAB32 (**J**) in “TCGA BRCA” compared with “TCGA normal” or “TCGA and GTEx normal” data

**Fig. 5:**
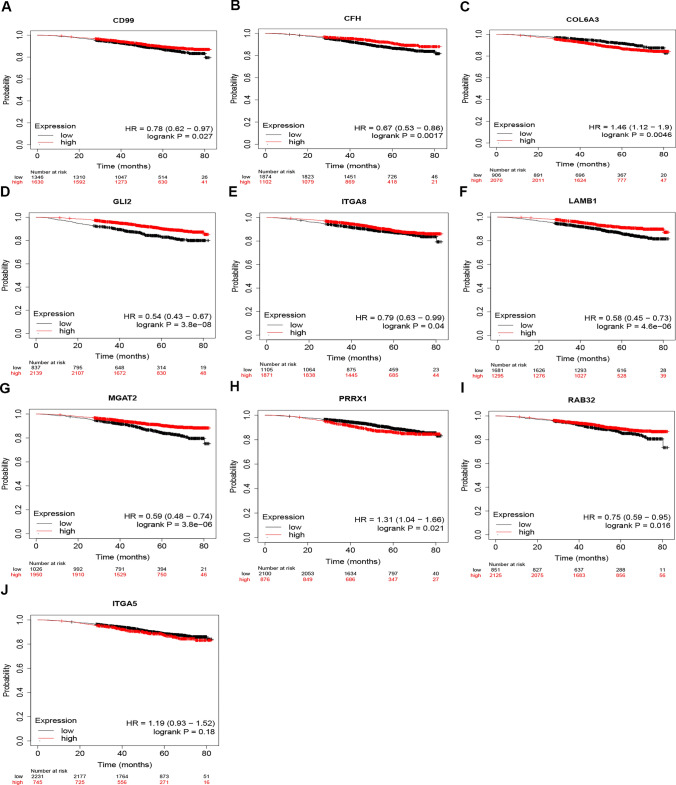
Survival analysis for upstream circRNAs of miR-30b-5p in BRCA. In BRCA the OS analysis for CD99 (**A**), CFH (**B**), COL6A3 (**C**), GLI2 (**D**), ITGA8 (**E**), LAMB1 (**F**), MGAT2 (**G**), PRRX1 (**H**) and RAB32 (**I**) and ITGA5 (**J**) in BRCA

The starBase database was then used to analyze the amounts of expression of these circRNAs in BRCA. Only CD99 was not significantly regulated in BRCA as compared to normal controls, as seen in Fig. 4. The prognostic values of the ten circRNAs in BRCA were then assessed. BRCA patients with higher levels of CFH, GLI2, RAB32, LAMB1, MGAT2, CD99 and ITGA8 expression had a better prognosis,while those of COL6A3 and PRRX1 had a worse one, as illustrated in Fig. 5.

Taking expression, survival, and correlation studies into account, CHF, GLI2, RAB32, LAMB1, MGAT2, ITGA8, COL6A3 and PRRX1 could be the eight most likely upstream circRNAs of the miR-30b-5p/PIK3CD axis in BRCA.

### PIK3CD correlates with EMT, migration and invasion in BRCA

**Fig. 6:**
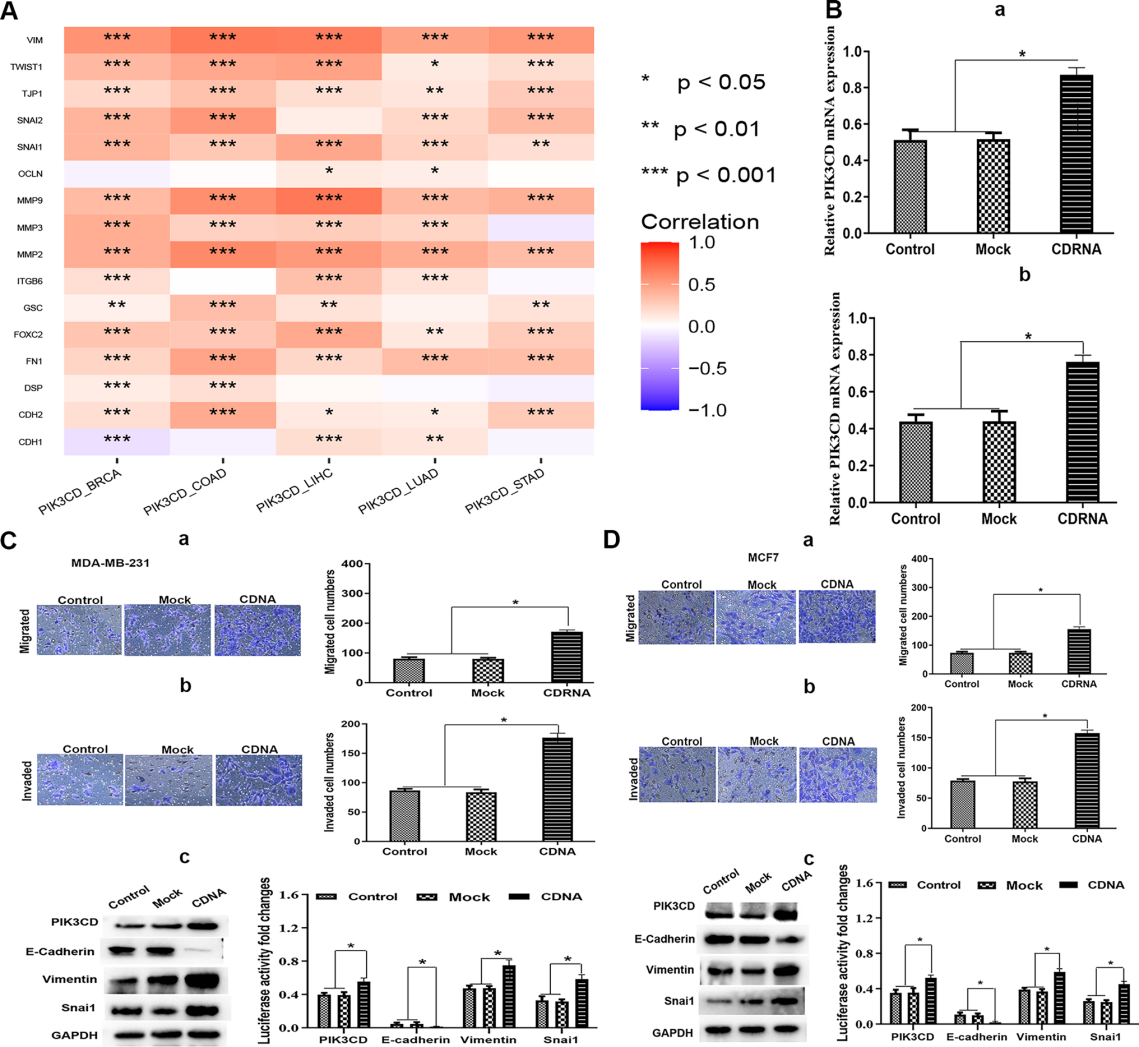
PIK3CD correlates with EMT in BRCA. **A** Correlation of PIK3CD expression with expression of EMT biomarkers across 5 cancer types. **B** The levels of PIK3CD mRNA in BRCA cell lines were detected by using qRT-PCR. a MDA-MB-231 cells. b MCF-7 cells. **C** Upregulation of PIK3CD increased MDA-MB-231 BRCA cell migration and invasion. Transwell assay of cell migration ability following PIK3CD cDNA treatment. Transwell assessment of cell invasion capabilities following PIK3CD cDNA transfection (a and b). (c) Western blot examination of EMT-related marker expression alterations following PIK3CD cDNA transfection. The statistical analysis was displayed. **D** Upregulation of PIK3CD enhanced MCF-7 BRCA cell motility and invasion. Transwell experiment of cell migration ability following PIK3CD cDNA treatment. Transwell test of cell invasion capabilities following PIK3CD cDNA transfection (a and b). (c) Western blot study of EMT-related marker expression following PIK3CD cDNA transfection. The statistical analysis was displayed

The link between PIK3CD and EMT variables was then investigated. In the majority of the 5 cancer types, PIK3CD expression was linked favorably with the expression of mesenchymal markers such as VIM (Vimentin), TWIST1, SNAI1 (SNAIL), SNAI2 (SLUG), and CDH2 (Fig. 6A). Furthermore, PIK3CD was found to be strongly inversely related to the epithelial marker CDH1 (E-Cadherin). The correlation heatmaps revealed a strong relationship between PIK3CD and the EMT variables. These findings showed that PIK3CD may be involved in EMT.

We studied the impact of PIK3CD on cell migration, invasion, and EMT of BRCA cells after verifying its expression and prognostic value for BRCA. MCF-7 BRCA cell lines were transfected with PIK3CD cDNA. To evaluate the effect of PIK3CD on cell migration and invasion, Transwell Assay was utilized. BRCA cells that received PIK3CD cDNA displayed higher migration and invasion abilities (Fig. 6B–D). After PIK3CD cDNA transfection, the expression of EMT-related markers was evaluated using Western blot. E-cadherin expression was downregulated in the PIK3CD cDNA group, while VIM and SNAI1 expression were elevated.

These findings demonstrated that PIK3CD cDNA increased cell EMT, migration, and invasion, indicating that PIK3CD plays an active role in BRCA cell activities.

### PIK3CD correlates with influx of immunological cells in BRCA

**Fig. 7:**
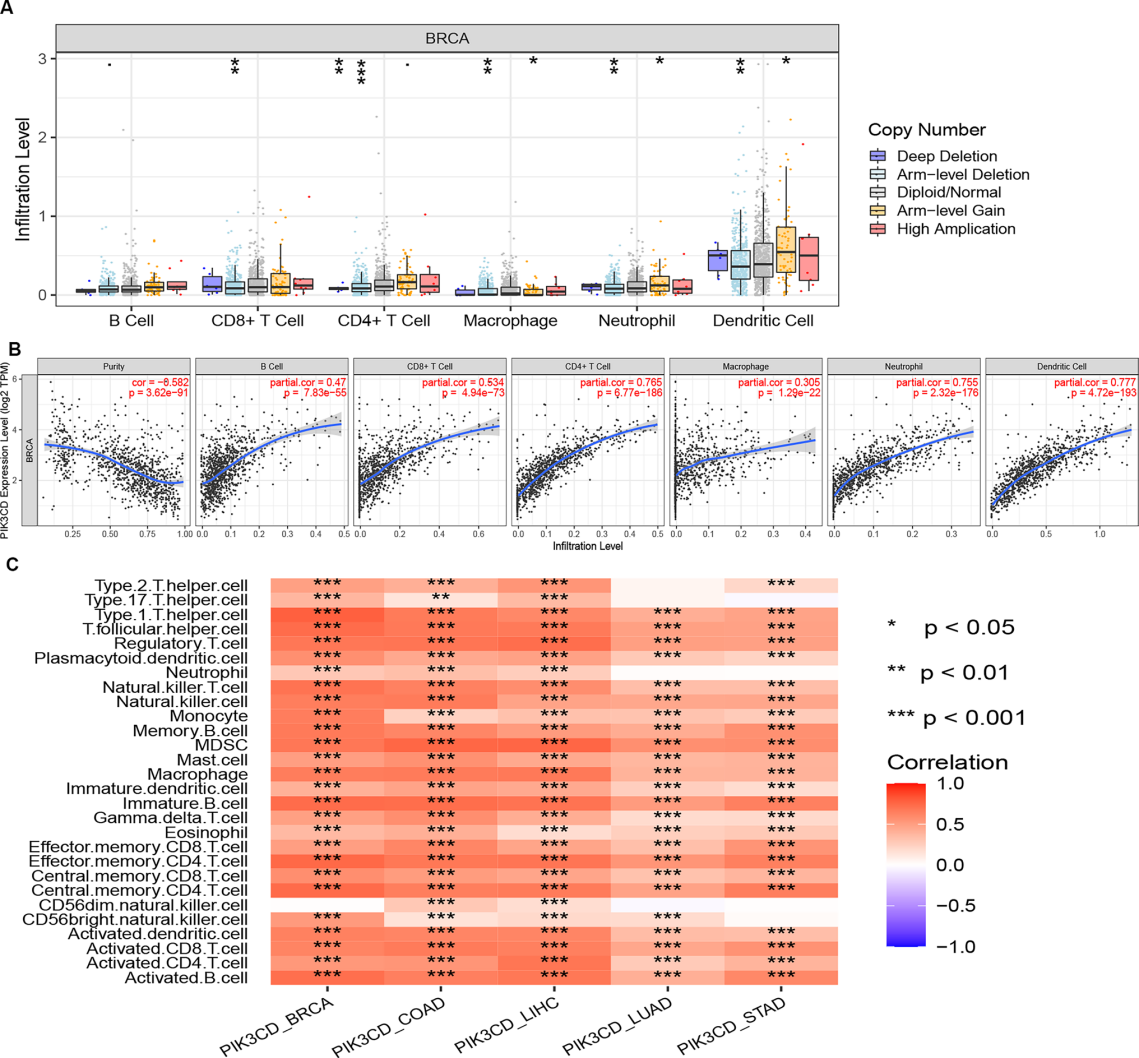
The association between immune cell infiltration and PIK3CD levels in BRCA. **A** The level of invasion of various immune cells in BRCA with varying copy levels of PIK3CD from the TIMER database. **B** The level of PIK3CD expression in BRCA is correlated with B cell (b), CD8 + T cell (c), CD4 + T cell (d), macrophage (e), neutrophil (f), or dendriticcell (g) infiltration. **C** Correlation of PIK3CD with immune cells 5 cancer types.The CIBERSORT technique was used in this work to estimate the fraction of 22 immune cells

The TIMER database was used to demonstrate the link between PIK3CD expression and immune cell infiltration levels in BRCA cells. The amount of immune cell infiltration significantly changed with different PIK3CD copy counts in BRCA, as illustrated in Fig. 7A. Somatic copy number alterations (SCNA) of PIK3CD were closely correlated with the abundance of immune infiltration. Consequently, the relationship between PIK3CD expression and immune cell infiltration was assessed. According to Fig. 7B, the expression of PIK3CD was highly positively linked with every immunological cell type that was investigated, including B cells,CD4 + T cells, CD8 + T cells,neutrophils, macrophages and dendritic cells in BRCA**.**

Clinical treatment sensitivity and disease diagnosis are significantly impacted by the microenvironment, which includes immune cells, extracellular matrix, inflammatory chemicals, and other growth factors. Using the CIBERSORT technique, the fraction of 22 immune cells was estimated using a histogram in this study. The results showed that the tumor group had considerably larger proportions of most immune cells than the control groups, with the exception of some immune cells such as CD56 dim natural killer cells in BRCA,LUAD, and STAD (Fig. 7C).

### Correlation of PIK3CD expression and immune cell indicators in BRCA

The GEPIA database was used to examine the expression connection of PIK3CD with immune cell indicators in BRCA, able to better investigate the function of PIK3CD in tumor immunity. According to the findings, PIK3CD expression was strongly positively linked with immunity-associated gene marker expression, such as biomarkers of B cells including CD19 and CD79A, and biomarkers of dendritic cells in BRCA (Table 4, *p value < 0.05; **p value < 0.01; ***p value < 0.001). These findings revealed that increased PIK3CD levels were related to increase immunological infiltration as well as immune exhaustion, suggesting that PIK3CD could be a potential immunotherapeutic target for BRCA treatment. These results help to some extent establish the favorable relationship between PIK3CD and immune cell infiltration.

**Table 4:** Correlation analysis between PIK3CD and biomarkers of immune cells in BRCA determined by GEPIA database

Immune cell	Biomarker	R value	p value
B cell	CD19	0.63	0***
	CD79A	0.62	0***
CD8 + T cell	CD8A	0.67	0***
	CD8B	0.54	0***
CD4 + T cell	CD4	0.65	0***
M1 macrophage	NOS2	− 0.0021	0.94
	IRF5	0.39	0***
	PTGS2	0.037	0.17
M2 macrophage	CD163	0.23	0***
	VSIG4	0.18	2.6E−11***
	MS4A4A	0.31	0***
	ITGAM	0.24	0***
	CCR7	0.33	0***
Dendritic cell	HLA-DPB1	0.61	0***
	HLA-DQB1	0.4	0***
	HLA-DRA	0.6	0***
	HLA-DPA1	0.56	0***
	CD1C	0.53	0***
	NRP1	0.12	6.2e−06***
	ITGAX	0.46	0***

*p value < 0.05; **p value < 0.01; ***p value < 0.001

### Link between PIK3CD and immunological checkpoints in BRCA

**Fig. 8:**
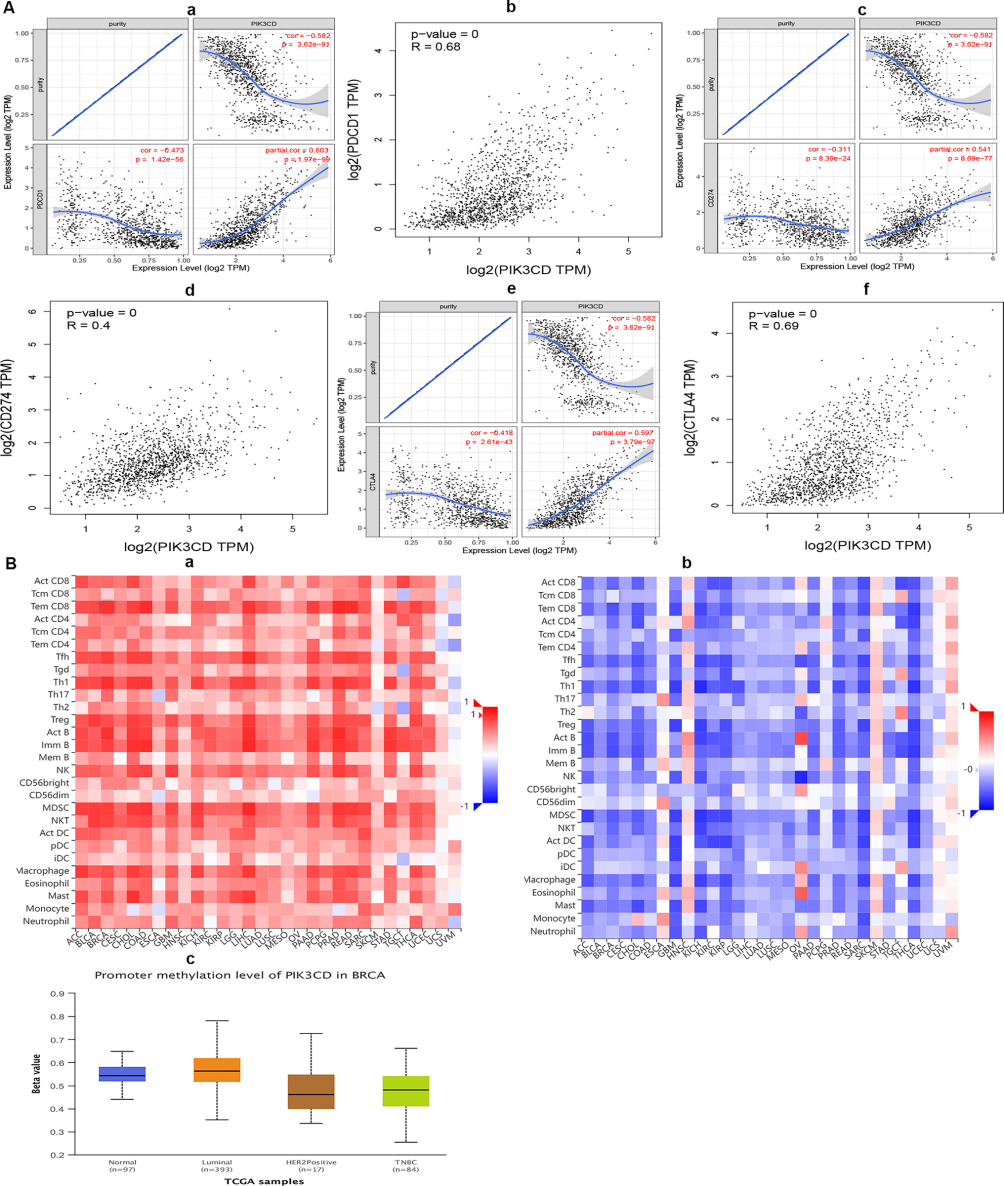
Relationship between PIK3CD and immune checkpoints/promoter methylation level of PIK3CD in BRCA. **A** Correlation of PIK3CD expression in BRCA with PD-1, PD-L1, and CTLA-4 expression. (a) TIMER-adjusted Spearman association of PIK3CD with PD-1 expression in BRCA. (b) The GEPIA database determined the expression connection of PIK3CD with PD1 in BRCA. (c) PIK3CD-PD-L1 TIMER-adjusted Spearman correlation in BRCA. (d) The GEPIA database determined the expression connection of PIK3CD with PD-L1 in BRCA. (e) TIMER Spearman association of PIK3CD with CTLA-4 expression in BRCA corrected for purity. (f) The GEPIA database determined the expression connection of PIK3CD with CTLA-4 in BRCA. **B** The correlations between PIK3CD expression, methylation level, and Tumor-infiltrating lymphocyte (TIL) quantity. (a) Relationships between 28 different types of tumor-infiltrating lymphocytes (TILs) and PIK3CD expression in several tumors. (b) Correlations between 28 different types of TILs and PIK3CD methylation in several tumors. (c) Correlations between the promoter methylation level of PIK3CD and the clinicopathological traits of BRCA patients. UALCAN database was used to examine the relative promoter methylation level of PIK3CD based on major subclasses

Immune checkpoints like PD1/PD-L1 and CTLA-4 are essential for tumor immunity escape. Given the putative oncogenic involvement of PIK3CD in BRCA, the association of PIK3CD with PD1, PD-L1,or CTLA-4 was investigated.As demonstrated in Fig. 8A, PIK3CD expression was strongly positively linked with PD1, PD-L1 and CTLA-4 in BRCA,which was modified by purity. We also found that PD1, PD-L1, CTLA-4 and PIK3CD in BRCA had a highly significant relationship, similar to what we found in the analysis of the TIMER data.Our findings show that PIK3CD-mediated tumorigenesis of BRCA may entail tumor immune evasion.

### Relationships between PIK3CD expression, methylation level and the amount of lymphocytes that infiltrate tumors

We discovered that PIK3CD expression had a positive relationship with the abundance of 28 different types of tumor-infiltrating lymphocytes in the TISIDB database (Fig. 8B, Table 5). We investigated the association between PIK3CD methylation and tumor-infiltrating lymphocyte abundance and discovered that it was strongly inversely linked with tumor-infiltrating lymphocyte abundance (Fig. 8B, Table 5). The results indicated that PIK3CD level was favorably related to the high number of lymphocytes infiltrating tumors, but PIK3CD methylation was negatively associated, and it might be used as one of the immunotherapeutic targets for BRCA therapy.

**Table 5:** Associations between PIK3CD expression/methylation level and TIL abundance

Cell name	Spearman correlation test: Rho	p value
The relationship between PIK3CD expression and TIL abundance		
Act_CD8	0.592	P < 2.2e−16
Tcm_CD8	0.193	P = 1.33e−10
Tem_CD8	0.825	P < 2.2e−16
Act_CD4	0.484	P < 2.2e−16
Tcm_CD4	0.441	P < 2.2e−16
Tem_CD4	0.388	P < 2.2e−16
Tfh	0.762	P < 2.2e−16
Tgd	0.53	P < 2.2e−16
Th1	0.815	P < 2.2e−16
Th17	0.592	P < 2.2e−16
Th2	0.389	P < 2.2e−16
Treg	0.701	P < 2.2e−16
Act_B	0.812	P < 2.2e−16
Imm_B	0.749	P < 2.2e−16
Mem_B	0.438	P < 2.2e−16
NK	0.566	P < 2.2e−16
CD56bright	0.481	P < 2.2e−16
CD56dim	0.324	P < 2.2e−16
MDSC	0.757	P < 2.2e−16
NKT	0.741	P < 2.2e−16
Act_DC	0.539	P < 2.2e−16
pDC	0.403	P < 2.2e−16
iDC	0.155	P = 2.62e−07
Macrophage	0.701	P < 2.2e−16
Eosinophil	0.592	P < 2.2e−16
Mast	0.69	P < 2.2e−16
Monocyte	0.474	P < 2.2e−16
Neutrophil	0.306	P < 2.2e−16
The relationship between PIK3CD methylation and TIL abundance		
Act_CD8	− 0.592	P < 2.2e−16
Tcm_CD8	− 0.188	P = 1.13e−07
Tem_CD8	− 0.676	P < 2.2e−16
Act_CD4	− 0.559	P < 2.2e−16
Tcm_CD4	− 0.39	P < 2.2e−16
Tem_CD4	− 0.394	P < 2.2e−16
Tfh	− 0.641	P < 2.2e−16
Tgd	− 0.434	P < 2.2e−16
Th1	− 0.66	P < 2.2e−16
Th17	− 0.428	P < 2.2e−16
Th2	− 0.349	P < 2.2e−16
Treg	− 0.569	P < 2.2e−16
Act_B	− 0.683	P < 2.2e−16
Imm_B	− 0.697	P < 2.2e−16
Mem_B	− 0.374	P < 2.2e−16
NK	− 0.447	P < 2.2e−16
CD56bright	− 0.402	P < 2.2e−16
CD56dim	− 0.258	P = 2.52e−13
MDSC	− 0.647	P < 2.2e−16
NKT	− 0.597	P < 2.2e−16
Act_DC	− 0.524	P < 2.2e−16
pDC	− 0.306	P = 2.05e−18
iDC	− 0.156	P = 1.1e−05
Macrophage	− 0.546	P < 2.2e−16
Eosinophil	− 0.495	P < 2.2e−16
Mast	− 0.529	P < 2.2e−16
Monocyte	− 0.372	P < 2.2e−16
Neutrophil	− 0.25	P = 1.56e−12

We also discovered links between PIK3CD methylation and clinicopathological characteristics in BRCA patients. In TNBC and HER2-Positive breast cancer tissues, the promoter methylation level of PIK3CD was lower than in Luminal (Fig. 8B). Thus, reduced PIK3CD methylation could be an underlying indication reflecting clinical BRCA features.

### Identification of the m1A/m5C/m6A regulators relevant to PIK3CD in BRCA

**Fig. 9:**
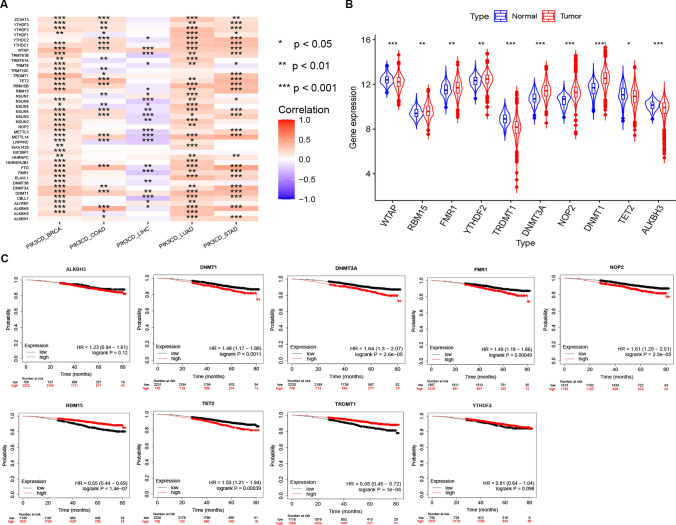
The m1A/m5C/m6A regulators relevant to PIK3CD in BRCA. **A** Relations between the levels of PIK3CD and the expressions of 41 kinds of the m1A/m5C/m6A regulators in various cancers. **B** The m1A/m5C/m6A regulator expression variations between tumor and normal samples. The vioplot shows the top 10 genes (|R|) that are significantly differentially expressed in breast tumors and normal tissues. **C** The overall survival (OS) analysis for the top 10 m1A/m5C/m6A regulators significantly differentially expressed (|R|) in both breast tumor and normal samples determined by Kaplan–Meier Plotter

The pan-cancer correlations between PIK3CD and the expression levels of 40 kinds of the m1A/m5C/m6A regulators in 5 cancer types were shown in Fig. 9A. There was a strong association (P < 0.05) between the expression levels of the 41 different m1A/m5C/m6A regulators and PIK3CD in 5 different cancer types. Furthermore,in BRCA and LUAD, significant co-expression of PIK3CD with most regulator genes was detected.In LIHC, expression of PIK3CD was negatively correlated with some molecules such as ZC3H13, YTHDF3 and TRMT61A, though for some of them not to a significant degree. It is also interesting that expression of PIK3CD was negatively correlated with TRMT61B, FMR1, FTO and METTL14 in LIHC,but the levels of PIK3CD were positively associated with them in BRCA (Fig. 9A).

In BRCA patients, DNMT1, NOP2, DNMT3A, FMR1, RBM15 and YTHDF2 were highly elevated, while WTAP, TET2, ALKBH3 and TRDMT1 were dramatically downregulated (Fig. 9B). For OS, high expression of ALKBH3, DNMT1, DNMT3A, FMR1, NOP2, TET2, acted as high-risk factors in BRCA and had unfavorable prognosis, but RBM15, TRDMT1 and WTAP indicated better prognosis (Fig. 9C).

## Discussion

BRCA is still known for having a terrible prognosis. The discovery of useful prognostic biomarkers or the creation of useful therapy targets may result from an understanding of the molecular process of BRCA. Numerous human cancers’ genesis and development have been shown to be significantly influenced by PIK3CD, among other things. However, our understanding of PIK3CD in BRCA remains limited and has to be expanded.

In this investigation, we first used data from The Cancer Genome Atlas (TCGA) and Genotype-Tissue Expression (GTEx) to do a pan-cancer analysis of PIK3CD expression.PIK3CD was significantly downregulated in 10 tumor types, including BRCA, COAD, LIHC, LUAD and LUSC (Fig. 1). As an intrinsic cancer-causing driver, PIK3CD was found in primary fibroblasts but was scarcely detectable in breast cancer cell lines [5]. According to the survival analysis for the cancer types of interest, patients with high PIK3CD expression had a favourable outlook for survival (OS) in LUAD and BRCA and a poor outcome in PRAD and LUSC. In individuals with laryngeal squamous cell carcinoma, PIK3CD expression was elevated and could predict a bad prognosis [6]. Fibroblast-expressed PIK3CD was a distinct predictive factor for overall and disease-free survival, indicating it as a therapeutic target for TNBC [5].

We used PicTar, RNA22, PITA, miRanda, microT, miRmap, and TargetScan to identify candidate miRNAs that may potentially bind to PIK3CD in order to examine the upstream regulatory miRNAs of PIK3CD. Six miRNAs were discovered in the end (Table 1). In laryngeal squamous cell carcinoma, the miR-30c-5p/PIK3CD axis is related to malignant properties and predicts a prognosis [6]. The bulk of these miRNAs have been identified as tumor-suppressive miRNAs in BRCA patients. Following correlation, expression, and survival analyses, miR-30b-5p was identified as the most likely upstream tumor suppressive miRNA of PIK3CD. It is reported that miR-30b-5p inhibits lung cancer cell growth and increases cisplatin sensitivity [7].

It has been extensively established that miRNAs and circRNAs contribute to gene expression regulation by communicating with one another via the ceRNA mechanism [8, 9]. Based on the ceRNA hypothesis, the putative circRNAs should be highly positively associated with the level of PIK3CD in BRCA.

Following that, upstream circRNAs of the miR-30b-5p/PIK3CD axis were predicted, and the top ten potential circRNAs(|R|) were identified. Eight of the most likely elevated circRNAs, including CFH, GLI2, RAB32, LAMB1, MGAT2, ITGA8, COL6A3 and PRRX1, were discovered using expression, survival, and correlation analysis.

These circRNAs, including COL6A3 [10], PRRX1 [11], CFH [12], GLI2 [13], RAB32 [14], LAMB1 [15], MGAT2 [16] and ITGA8 [17] have been implicated in a number of malignancies as oncogenes. Taken together, COL6A3, PRRX1, CFH, GLI2, RAB32, LAMB1, MGAT2 and ITGA8 / miR-30b-5p/PIK3CD axis were discovered as possible regulatory mechanisms in BRCA.

PIK3CD expression correlated favorably with the expression of mesenchymal markers and was strongly inversely related to the epithelial marker. The correlation heatmaps revealed a strong relationship between PIK3CD and the EMT variables. PIK3CD cDNA increased cell EMT, migration, and invasion, indicating that PIK3CD played an active role in BRCA cell activities (Fig. 6). These findings showed that PIK3CD may be involved in EMT. EMT was associated with immune infiltration [18] and EMT-related genes were related to prognosis. PIK3CD significantly boosted colorectal cancer cell motility, invasion, and proliferation in vitro, as well as tumor development in vivo [2].

To our interest, PIK3CD enhanced the migration, invasion, and EMT of BRCA cells,but PIK3CD might be used as a favorable prognostic biomarker in BRCA patients. PIK3CD was shown to be related to the expression of SNAI1. Luminal A tumors with high SNAI1 expression had a higher OS marker, while luminal B, Her2-positive, and basal-like malignancies had a poorer OS marker [19]. As a result, we hypothesized that PIK3CD was associated with a favorable prognostic biomarker for OS in the luminal A subtype. The most common subtype of breast cancer is luminal/estrogen receptor-positive (ER +) breast cancer, which includes luminal A and luminal B subtypes) [20].

Several studies have shown that immune cell infiltration in tumors can affect the efficacy of chemotherapy [21], radiotherapy [22], or immunotherapy [23], as well as cancer patients' prognosis [24]. PIK3CD correlated with immune cell infiltration in BRCA (Figs. 7–9, Table 4). In BRCA a substantial positive relationship was seen between PIK3CD and B cells, CD8 + T cells, CD4 + T cells, macrophages, neutrophils, and dendritic cells (Fig. 7B, C, Table 4). Furthermore, PIK3CD was found to be significantly related to biomarkers of these infiltrated immune cells. These results suggested that tumor immune infiltration might contribute to PIK3CD-mediated carcinogenic functions in BRCA.

The effectiveness of immunotherapy is also influenced by the expression of immunological checkpoints and the number of immune cells entering the tumor microenvironment [25]. As a result, we investigated the link between PIK3CD and immunological checkpoints. The findings showed that high PIK3CD expression was highly connected to PD1, PD-L1, or CTLA-4 in BRCA, implying that highly expressed PIK3CD may improve the efficacy of immunotherapy in BRCA (Fig. 8A).

We discovered that PIK3CD expression had a positive relationship with the abundance of 28 different types of tumor-infiltrating lymphocytes and that PIK3CD methylation was strongly inversely linked with tumor-infiltrating lymphocyte abundance in the TISIDB database. We also discovered links between PIK3CD methylation and clinicopathological characteristics in BRCA patients (Fig. 8B, Table 5). Reduced PIK3CD methylation could be an underlying indication reflecting clinical BRCA features. Tumor infiltrating lymphocytes (TIL) are critical in influencing treatment response and enhancing clinical outcomes in all subtypes of breast cancer [26]. Both HER2-positive and TNBCs have a better prognosis and treatment response when there are many of tumor-infiltrating lymphocytes (TILs) [27]. Tumors with low T cell infiltration are more resistant to immunogenic chemotherapies and checkpoint inhibition than cancers with high T cell infiltration [28]. Recently, DNA methylation profiles were discovered to be an indication of the tumor immune microenvironment [29]. PIK3CD expression was found to be favorably related to the infiltration of immune cells and gene-marker levels, which could be linked to methylation-related changes.

RNA modifications such as m6A, m5C and m1A have been shown in recent years to influence the immune response and cancer [30]. Correlation analyses found a high correlation between PIK3CD and the m1A/m5C/m6A regulators in 5 cancer types (Fig. 9). In hepatocellular carcinoma, m6A/m5C/m1A regulated genes were linked to a poor prognosis and an immunological microenvironment [31].

In the present study, we systematically investigated PIK3CD's function in BRCA. We identified that PIK3CD had the potential to operate as a detection and prognosis biomarker for BRCA by examining its underlying mechanism. We found an upstream regulatory mechanism for PIK3CD in BRCA, which included GLI2, RAB32, LAMB1, MGAT2, ITGA8, CHF, COL6A3 and PRRX1/miR-30b-5p/PIK3CD. Furthermore, our current findings suggest that PIK3CD may have an important function by boosting EMT, immune checkpoint expression and tumor immune cell infiltration. However, these results require confirmation in the future through much more fundamental research and substantial clinical trials. To date, there have been relatively few studies on PIK3CD in BRCA. Our comprehensive examination of PIK3CD and its conceivable mechanisms may serve as the basis for further investigation.

## Data Availability

Publicly available datasets were analyzed in this study.This data can be found here: The datasets analyzed for this study were obtained from The Cancer Genome Atlas(TCGA, https://portal.gdc.cancer.gov/), Genotype-Tissue Expression (GTEx) data and the UCSC Xena browser (https://xenabrowser.net).

## Abbreviations

BLCA: Bladder urothelial carcinoma
BRCA: Breast invasive carcinoma
CHOL: Cholangiocarcinoma
COAD: Colon adenocarcinoma
ESCA: Esophageal carcinoma
GBMLGG: Glioma
HNSC: Head and Neck squamous cell carcinoma
KICH: Kidney chromophobe
KIRC: Kidney renal clear cell carcinoma
KIRP: Kidney renal papillary cell carcinoma
LAML: Acute myeloid leukemia
LIHC: Liver hepatocellular carcinoma
LUAD: Lung adenocarcinoma
LUSC: Lung squamous cell carcinoma
OV: Ovarian serous cystadenocarcinoma
PAAD: Pancreatic adenocarcinoma
PRAD: Prostate adenocarcinoma
STAD: Stomach adenocarcinoma
SKCM: Skin cutaneous melanoma
STES: Stomach and esophageal carcinoma
TGCT: Testicular germ cell tumors
THCA: Thyroid carcinoma
UCEC: Uterine corpus endometrial carcinoma
WT: High-risk wilms tumor
EMT: Epithelial–mesenchymal transition
TIL: Tumor-infiltrating lymphocyte
TILs: Tumor-infiltrating lymphocytes
OS: Overall survival
RFS: Relapse-free survival
VIM: Vimentin

## References

[1] Sun YS, Zhao Z, Yang ZN, Xu F, Lu HJ, Zhu ZY, Shi W, Jiang J, Yao PP, Zhu HP (2017). Risk factors and preventions of breast cancer. Int J Biol Sci.

[2] Chen JS, Huang JQ, Luo B, Dong SH, Wang RC, Jiang ZK, Xie YK, Yi W, Wen GM, Zhong JF (2019). PIK3CD induces cell growth and invasion by activating AKT/GSK-3β/β-catenin signaling in colorectal cancer. Cancer Sci.

[3] Chae YK, Chang S, Ko T, Anker J, Agte S, Iams W, Choi WM, Lee K, Cruz M (2018). Epithelial–mesenchymal transition (EMT) signature is inversely associated with T-cell infiltration in non-small cell lung cancer (NSCLC). Sci Rep.

[4] Liu Z, Wang T, She Y (2021). N6-methyladenosine-modified circIGF2BP3 inhibits CD8+ T-cell responses to facilitate tumor immune evasion by promoting the deubiquitination of PD-L1 in non-small cell lung cancer. Mol Cancer..

[5] Gagliano T, Shah K, Gargani S, Lao L, Alsaleem M, Chen J, Ntafis V, Huang P, Ditsiou A, Vella V, Yadav K, Bienkowska K, Bresciani G, Kang K, Li L, Carter P, Benstead-Hume G, O'Hanlon T, Dean M, Pearl FM, Lee SC, Rakha EA, Green AR, Kontoyiannis DL, Song E, Stebbing J, Giamas G (2020). PIK3Cδ expression by fibroblasts promotes triple-negative breast cancer progression. J Clin Invest.

[6] Li X, Xu F, Meng Q, Gong N, Teng Z, Xu R, Zhao M, Xia M (2020). Long noncoding RNA DLEU2 predicts a poor prognosis and enhances malignant properties in laryngeal squamous cell carcinoma through the miR-30c-5p/PIK3CD/Akt axis. Cell Death Dis.

[7] Qiu H, Shen X, Chen B, Chen T, Feng G, Chen S, Feng D, Xu Q (2021). miR-30b-5p inhibits cancer progression and enhances cisplatin sensitivity in lung cancer through targeting LRP8. Apoptosis.

[8] Cheng Y, Su Y, Wang S, Liu Y, Jin L, Wan Q, Liu Y, Li C, Sang X, Yang L, Liu C, Wang Z (2020). Identification of circRNA-lncRNA-miRNA-mRNA competitive endogenous rna network as novel prognostic markers for acute myeloid leukemia. Genes (Basel).

[9] Zhu J, Zhang X, Gao W, Hu H, Wang X, Hao D (2019). lncRNA/circRNA-miRNA-mRNA ceRNA network in lumbar intervertebral disc degeneration. Mol Med Rep.

[10] Wei LY, Zhang XJ, Wang L, Hu LN, Zhang XD, Li L, Gao JN (2020). A six-epithelial–mesenchymal transition gene signature may predict metastasis of triple-negative breast cancer. Onco Targets Ther.

[11] Dong J, Lv Z, Chen Q, Wang X, Li F (2018). PRRX1 drives tamoxifen therapy resistance through induction of epithelial–mesenchymal transition in MCF-7 breast cancer cells. Int J Clin Exp Pathol.

[12] Hwang N, Chung SW (2020). Sulfasalazine attenuates tamoxifen-induced toxicity in human retinal pigment epithelial cells. BMB Rep.

[13] Johnson RW, Nguyen MP, Padalecki SS, Grubbs BG, Merkel AR, Oyajobi BO, Matrisian LM, Mundy GR, Sterling JA (2011). TGF-beta promotion of Gli2-induced expression of parathyroid hormone-related protein, an important osteolytic factor in bone metastasis, is independent of canonical Hedgehog signaling. Cancer Res.

[14] Zhang Y, Zhou M, Li K (2022). MicroRNA-30 inhibits the growth of human ovarian cancer cells by suppressing RAB32 expression. Int J Immunopathol Pharmacol..

[15] Lee H, Kim WJ, Kang HG, Jang JH, Choi IJ, Chun KH, Kim SJ (2021). Upregulation of LAMB1 via ERK/c-Jun axis promotes gastric cancer growth and motility. Int J Mol Sci.

[16] Hall MK, Burch AP, Schwalbe RA (2021). Functional analysis of N-acetylglucosaminyltransferase-I knockdown in 2D and 3D neuroblastoma cell cultures. PLoS ONE.

[17] Wu J, Cheng J, Zhang F, Luo X, Zhang Z, Chen S (2020). Estrogen receptor α is involved in the regulation of ITGA8 methylation in estrogen receptor-positive breast cancer. Ann Transl Med.

[18] Kong W, Mao Z, Han C, Ding Z, Yuan Q, Zhang G, Li C, Wu X, Chen J, Guo M, Hong S, Yu F, Liu R, Wang X, Zhang J (2022). A novel epithelial–mesenchymal transition gene signature correlated with prognosis, and immune infiltration in hepatocellular carcinoma. Front Pharmacol.

[19] Voutsadakis IA (2015). The network of pluripotency, epithelial–mesenchymal transition, and prognosis of breast cancer. Breast Cancer (Dove Med Press).

[20] Hernando C, Ortega-Morillo B, Tapia M, Moragón S, Martínez MT, Eroles P, Garrido-Cano I, Adam-Artigues A, Lluch A, Bermejo B, Cejalvo JM (2021). Oral selective estrogen receptor degraders (SERDs) as a novel breast cancer therapy: present and future from a clinical perspective. Int J Mol Sci.

[21] Kim R, An M, Lee H, Mehta A, Heo YJ, Kim KM, Lee SY, Moon J, Kim ST, Min BH, Kim TJ, Rha SY, Kang WK, Park WY, Klempner SJ, Lee J (2022). Early tumor-immune microenvironmental remodeling and response to first-line fluoropyrimidine and platinum chemotherapy in advanced gastric cancer. Cancer Discov.

[22] Herrera FG, Ronet C, Ochoa de Olza M, Barras D, Crespo I, Andreatta M, Corria-Osorio J, Spill A, Benedetti F, Genolet R, Orcurto A, Imbimbo M, Ghisoni E, Navarro Rodrigo B, Berthold DR, Sarivalasis A, Zaman K, Duran R, Dromain C, Prior J, Schaefer N, Bourhis J, Dimopoulou G, Tsourti Z, Messemaker M, Smith T, Warren SE, Foukas P, Rusakiewicz S, Pittet MJ, Zimmermann S, Sempoux C, Dafni U, Harari A, Kandalaft LE, Carmona SJ, Dangaj Laniti D, Irving M, Coukos G (2022). Low-dose radiotherapy reverses tumor immune desertification and resistance to immunotherapy. Cancer Discov..

[23] Zhang Y, Zhang Z (2020). The history and advances in cancer immunotherapy: understanding the characteristics of tumor-infiltrating immune cells and their therapeutic implications. Cell Mol Immunol.

[24] Zou Q, Wang X, Ren D, Hu B, Tang G, Zhang Y, Huang M, Pai RK, Buchanan DD, Win AK, Newcomb PA, Grady WM, Yu H, Luo Y (2021). DNA methylation-based signature of CD8+ tumor-infiltrating lymphocytes enables evaluation of immune response and prognosis in colorectal cancer. J Immunother Cancer.

[25] Feng M, Jiang W, Kim BYS, Zhang CC, Fu YX, Weissman IL (2019). Phagocytosis checkpoints as new targets for cancer immunotherapy. Nat Rev Cancer.

[26] Stanton SE, Disis ML (2016). Clinical significance of tumor-infiltrating lymphocytes in breast cancer. J Immunother Cancer.

[27] González-Martínez S, Pérez-Mies B, Pizarro D, Caniego-Casas T, Cortés J, Palacios J (2021). Epithelial mesenchymal transition and immune response in metaplastic breast carcinoma. Int J Mol Sci.

[28] Bruchard M, Geindreau M, Perrichet A, Truntzer C, Ballot E, Boidot R, Racoeur C, Barsac E, Chalmin F, Hibos C, Baranek T, Paget C, Ryffel B, Rébé C, Paul C, Végran F, Ghiringhelli F (2022). Recruitment and activation of type 3 innate lymphoid cells promote antitumor immune responses. Nat Immunol.

[29] Xu B, Lu M, Yan L, Ge M, Ren Y, Wang R, Shu Y, Hou L, Guo H (2021). A pan-cancer analysis of predictive methylation signatures of response to cancer immunotherapy. Front Immunol.

[30] Qi L, Zhang W, Ren X, Xu R, Yang Z, Chen R, Tu C, Li Z (2022). Cross-talk of multiple types of RNA modification regulators uncovers the tumor microenvironment and immune infiltrates in soft tissue sarcoma. Front Immunol.

[31] Li D, Li K, Zhang W, Yang KW, Mu DA, Jiang GJ, Shi RS, Ke D (2022). The m6A/m5C/m1A regulated gene signature predicts the prognosis and correlates with the immune status of hepatocellular carcinoma. Front Immunol.

